# Myoinhibitory peptide signaling modulates aversive gustatory learning in *Caenorhabditis elegans*

**DOI:** 10.1371/journal.pgen.1007945

**Published:** 2019-02-19

**Authors:** Katleen Peymen, Jan Watteyne, Charline Borghgraef, Elien Van Sinay, Isabel Beets, Liliane Schoofs

**Affiliations:** Department of Biology, University of Leuven (KU Leuven), Leuven, Belgium; Stanford University School of Medicine, UNITED STATES

## Abstract

Aversive learning and memories are crucial for animals to avoid previously encountered stressful stimuli and thereby increase their chance of survival. Neuropeptides are essential signaling molecules in the brain and are emerging as important modulators of learned behaviors, but their precise role is not well understood. Here, we show that neuropeptides of the evolutionarily conserved MyoInhibitory Peptide (MIP)-family modify salt chemotaxis behavior in *Caenorhabditis elegans* according to previous experience. MIP signaling, through activation of the G protein-coupled receptor SPRR-2, is required for short-term gustatory plasticity. In addition, MIP/SPRR-2 neuropeptide-receptor signaling mediates another type of aversive gustatory learning called salt avoidance learning that depends on *de novo* transcription, translation and the CREB transcription factor, all hallmarks of long-term memory. MIP/SPRR-2 signaling mediates salt avoidance learning in parallel with insulin signaling. These findings lay a foundation to investigate the suggested orphan MIP receptor orthologs in deuterostomians, including human GPR139 and GPR142.

## Author summary

All animals rely on learning and memory processes to learn from experience and thereby increase their chance of survival. Neuropeptides are essential signaling molecules in the brain and are emerging as important modulators of learning and memory processes. We found that the *C*. *elegans* receptor SPRR-2 and its ligands, the MIP-1 neuropeptides—which are members of the evolutionarily conserved myoinhibitory peptide system—are required for aversive gustatory learning. Our results provide a basis for investigations into the poorly characterized MIP systems in deuterostomians, including humans, and suggest a possible function in learning for human MIP signaling.

## Introduction

In a dynamic environment animals have to adapt their choices and behavioral responses according to previous experiences to increase their chances of survival. Therefore, animals evolved the ability to learn and generate memories through associative and non-associative neural mechanisms [1,2]. Knowledge on the molecular pathways underlying learning and memory is essential to uncover the complex regulation of experience-dependent plasticity in neural circuits and its decline with age or in associated diseases. Early studies in invertebrate model systems, such as *Aplysia*, have been vital to our current knowledge on the molecular basis of learning and memory [1,3]. Later studies in *Drosophila* and rodents revealed that the molecular pathways underlying memory storage seem to be evolutionarily conserved [3,4]. More recently, *Caenorhabditis elegans* has become a popular model for uncovering genes and mechanisms of circuit plasticity that regulate learning and memory [2,5]. The nematode has a small nervous system of 302 neurons, the synaptic connections of which have nearly all been mapped [6]. Despite its anatomical simplicity, *C*. *elegans* shows associative and non-associative learning in response to a variety of sensory cues and can form both short-term and long-term memories [2,7].

One example of a learned behavior in *C*. *elegans* is the food-dependent modulation of salt chemotaxis behavior. Exposure to a certain NaCl concentration in the presence of food shifts the nematode’s salt preference towards this concentration [8]. In contrast, pairing NaCl with starvation for several hours induces gustatory aversive learning, referred to as salt avoidance learning [8–10]. Short-term conditioning with NaCl in the absence of food also induces aversive learning, known as gustatory plasticity [11–13].

We previously found that short-term gustatory plasticity in *C*. *elegans* is modulated by nematocin, a neuropeptide of the vasopressin-oxytocin family [11]. Also other neuropeptides have been associated with learning and memory in a variety of animal species [14–17]. Neuropeptides are an evolutionarily ancient and diverse class of neural messengers that mainly act through binding of G protein-coupled receptors (GPCRs) and are ubiquitously involved in animal physiology and behavior [16–21]. The hypothesis that neuropeptides are crucial regulators of learned behaviors is an emerging trend and all animals capable of learning harbor a plethora of neuropeptide-encoding genes. However, modulation of the various types of learning and memory processes by the broad repertoire (hundreds) of neuropeptides in the central nervous system (CNS) seems to be highly complex and gaining insight into the roles of specific neuropeptides in learning circuits has proven to be difficult, especially in mammals [14]. In addition to ~150 genes for neuropeptide GPCRs, many of which are evolutionarily conserved [11,20,21], the *C*. *elegans* genome encodes at least 153 neuropeptide precursors that can generate over 300 bioactive peptides [21,22].

Using a candidate gene approach, we sought to identify neuropeptides and neuropeptide GPCRs that regulate associative learning in *C*. *elegans* by performing gustatory learning tests. We focused on neuropeptides that are conserved across the Animal Kingdom and of which the cellular expression is largely observed in brain areas known to be involved in learning and memory. Such a neuropeptide system is the evolutionarily conserved myoinhibitory peptide (MIP) system, initially discovered in locusts and mainly studied in insects [23–26]. Although MIP neuropeptides and their receptors have not been directly implicated in learning, we hypothesized that they may modulate learning circuits based on their expression in the insect mushroom body, an area of the CNS that is crucial for olfactory and gustatory conditioning in insects [26].

*C*. *elegans* has three predicted MIP receptor orthologs: SPRR-1, SPRR-2, and SPRR-3 (*s*ex *p*eptide *r*eceptor *r*elated [27–29]), belonging to the rhodopsin class of GPCRs. Here, we found that MIP signalling modulates aversive learning in *C*. *elegans*. We discovered that by activation of SPRR-2, MyoInhibitory Peptides encoded by the MIP-1 neuropeptide precursor promote two types of gustatory associative learning in *C*. *elegans*, i.e. gustatory plasticity and salt avoidance learning. We found that salt avoidance learning depends on similar molecular mechanisms as long-term memory in other organisms, including *de novo* transcription, translation and CREB activation, and that *sprr-2* modulates salt avoidance learning in parallel to the insulin signaling pathway.

The study of avoidance learning has recently regained interest both in experimental psychology research and in clinical psychology, because of its key role in normal psychological functioning as well as in mental disorders, such as Obsessive Compulsive or Post-Traumatic Stress Disorder. Yet, our understanding of the underlying neural and molecular mechanisms is scarce [30]. Because 17 years after the completion of the human genome sequence the suggested MIP receptor orthologs in mammals, known as GPR139 and GPR142, are still orphan and poorly explored [27,28,31], these findings lay a foundation for further research on the involvement of MIP receptor signaling in avoidance learning in other organisms, including humans.

## Results

### The *C*. *elegans* MIP receptor ortholog SPRR-2 regulates short-term gustatory plasticity

**Fig 1:**
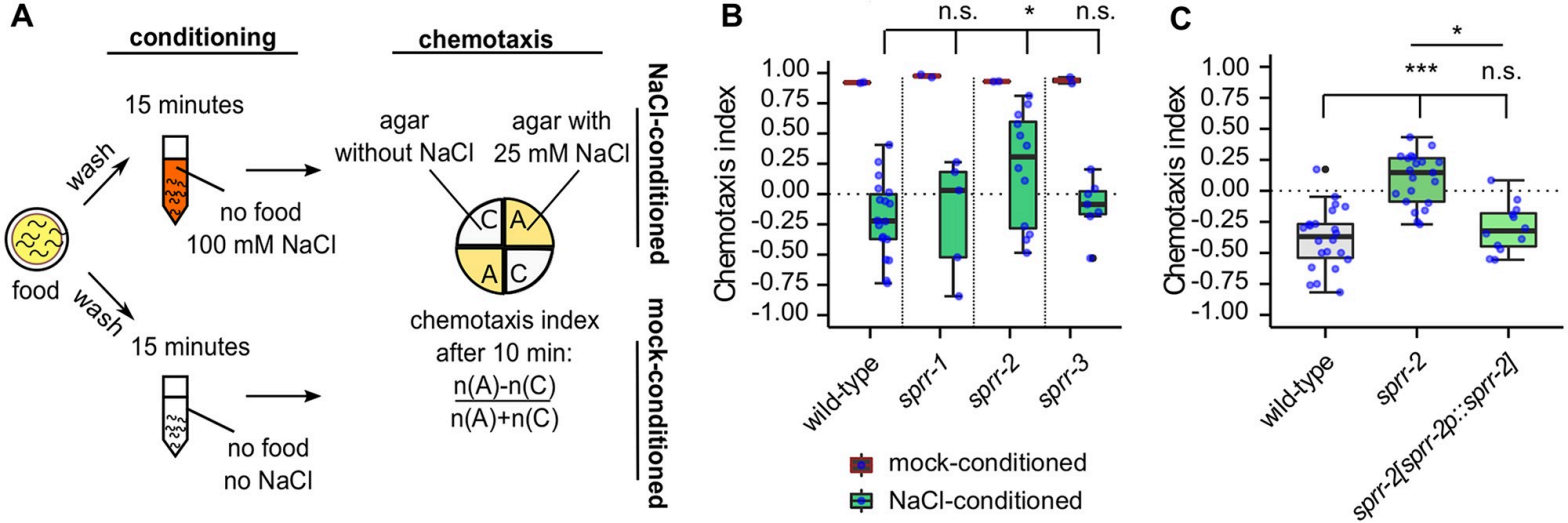
MIP receptor signaling promotes short-term gustatory plasticity. (A) Overview of the gustatory plasticity assay: Short-term conditioning with NaCl in the absence of food induces aversive learning, known as gustatory plasticity [11,12]. Synchronized young adult *C*. *elegans* are washed in buffer in the absence of food with salt (NaCl-conditioned) or without NaCl (mock-conditioned). Chemotaxis behavior to NaCl is then tested on a quadrant plate. After ten minutes, a chemotaxis index (CI) is calculated as indicated. (B-C) Gustatory plasticity of *sprr* mutants. Individual CIs are plotted as blue dots. Boxplots indicate 25^th^ (lower boundary), 50^th^ (line), and 75^th^ (upper boundary) percentiles. Whiskers show the minimum and maximum values. Outliers are indicated as black dots. (B) Comparison of CIs for mock-conditioned animals yielded p>0.05 for all genotypes (not indicated on graph). NaCl-conditioned mutants of *sprr-1* and *sprr-3* displayed wild-type avoidance of NaCl, whereas the response of *sprr-2* was significantly reduced. Data were analyzed by one-way ANOVA and Tukey post-hoc test (n ≥ 4). (C) The MIP receptor gene *sprr-2* is required for gustatory plasticity. Expression of *sprr-2* cDNA under the control of its promoter sequence [*sprr-2p*::*sprr-2*] rescues the plasticity defect of NaCl-conditioned *sprr-2* mutant animals. Statistical comparisons by one-way ANOVA and Tukey post-hoc test (n ≥ 12). *p<0.05; ***p<0.001; n.s., not significant. See also S1 and S2 Figs.

To test our hypothesis that MIP signaling mediates learning in *C*. *elegans*, we examined loss-of-function mutants of the three MIP receptor orthologs (*s*ex-*p*eptide-*r*eceptor*-r*elated or *sprr* genes) for their performance in an established associative learning test for gustatory plasticity [11–13]. In this test, worms are trained to avoid a normally attractive NaCl concentration by pairing NaCl as a conditioned stimulus with the unconditioned stimulus of short-term food withdrawal, similar to Pavlovian conditioning. To bring about this switch in salt chemotaxis behavior from NaCl attraction to salt aversion, animals were soaked in a NaCl-containing buffer without food during a 15-minute conditioning period (NaCl-conditioning), which translates into a negative chemotaxis index (Fig 1A and 1B). Wild-type *C*. *elegans* treated with a NaCl-free buffer (mock-conditioning) do not show this switch in salt chemotaxis behavior and show positive chemotaxis towards NaCl (Fig 1B).

Similar to wild-type animals, null mutants of *sprr-1* and *sprr-3* learn to avoid salt after NaCl-conditioning (Fig 1B). However, loss-of-function mutants of *sprr-2* are defective in gustatory plasticity, as they still show attraction towards NaCl after conditioning with salt in the absence of food (Fig 1B). Expressing wild-type copies of *sprr-2* under the control of its endogenous promoter fully restored the learning defect of the *sprr-2* mutant (Fig 1C).

The reduced gustatory plasticity of *sprr-2* loss-of-function mutants can be due to the fact that these animals are deficient in learning or might be caused by a general defect in neural circuits for movement or salt sensing. To distinguish between these possibilities, we quantified locomotion speed of *sprr-2* mutant animals and found that they crawl with a speed similar to that of wild-type animals on and off food (S1A and S1B Fig). Thus, the observed gustatory plasticity defect does not result from a general defect in locomotion. Furthermore, mock-conditioned *sprr-2* animals show normal NaCl chemotaxis behavior, both in the gustatory plasticity assay (Fig 1B) as well as to increasing NaCl concentrations (S1E Fig, see Methods). This indicates that primary functions for salt sensing are not affected in these mutants. When *sprr-2* mutants were conditioned for 30 minutes on NaCl-containing plates seeded with *E*. *coli* OP50 (S2A Fig), they displayed strong attraction to NaCl similar to wild-type animals (S2B Fig). This indicates that NaCl chemotaxis behavior is also unaffected in mutant animals pre-exposed to NaCl in the presence of food. Taken together, these results show that *sprr-2* is required for gustatory aversive learning.

**Fig 2:**
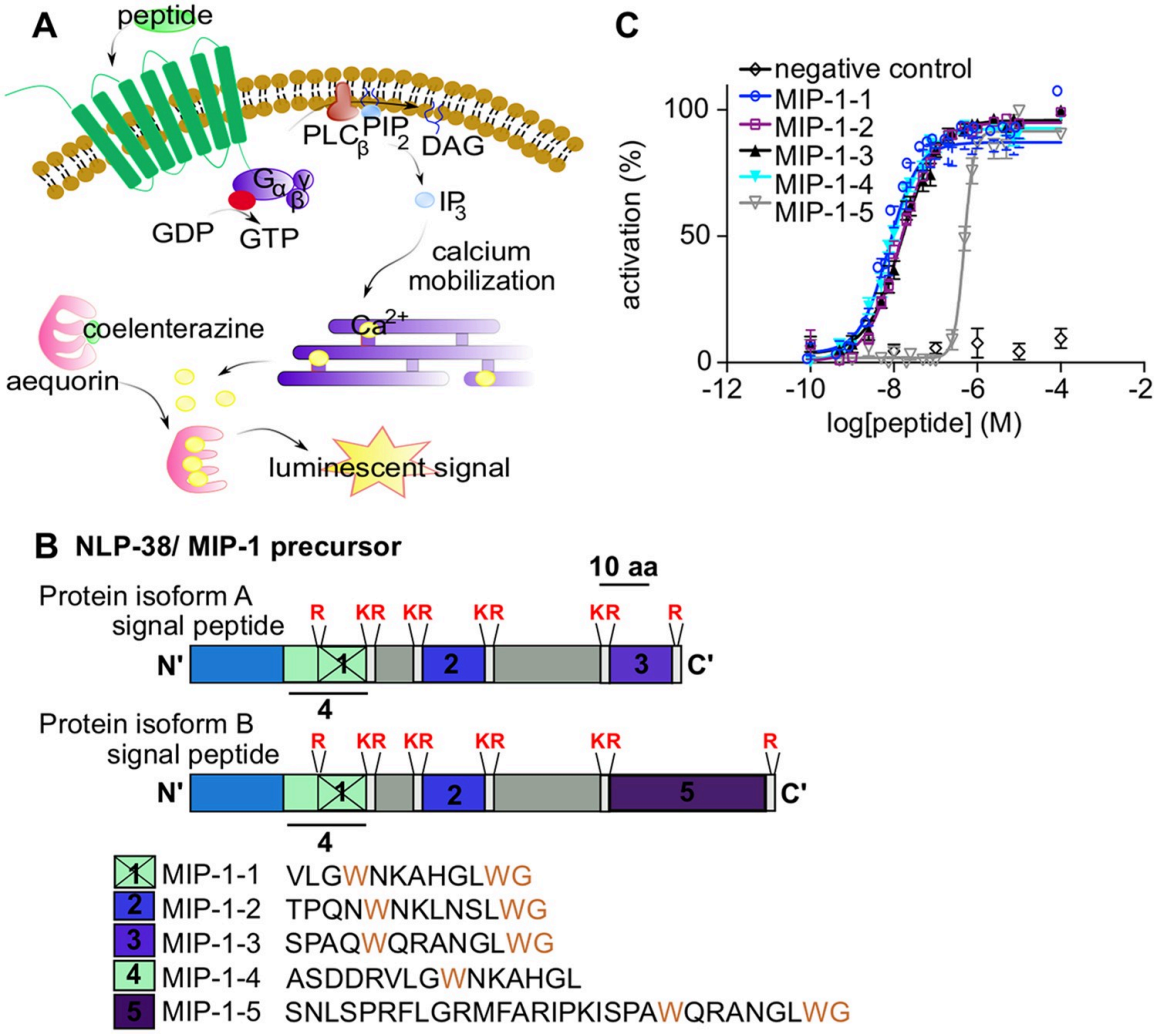
MIP-1 neuropeptides activate the MIP receptor ortholog SPRR-2. (A) Ca^2+^ luminescence assay for measuring GPCR activation. SPRR-2 is expressed in CHO cells that stably co-express the promiscuous G_α16_ protein, which couples the receptor’s activation to the Ca^2+^ pathway. Intracellular Ca^2+^ levels are monitored by the luminescent Ca^2+^ indicator aequorin. (B) Domain structure of the *C*. *elegans* NLP-38 peptide precursor, henceforward named MIP-1. Gene models predict that the *mip-1* gene encodes two protein isoforms (Wormbase WS261), MIP-1A and MIP-1B, which yield five putative MIP-1 peptides. Predicted proprotein convertase sites are indicated in red. Residues belonging to the W-X_5-8_-Wamide motif, typical of protostomian MIPs, are indicated in brown. (C) MIP-1 peptides dose-dependently activate SPRR-2. Ca^2+^responses of CHO cells expressing SPRR-2 are shown relative (%) to the highest value (100% activation) after normalization to the total Ca^2+^ response. EC_50_ values (95% CI) for MIP-1-1 to MIP-1-5 are 7.50 (6.21–9.01) nM, 14.19 (12.64–15.90) nM, 16.07 (13.65–18.91) nM, 8.32 (7.16–9.64) nM, and 486.41 (455.60–520.00) nM. Error bars show SEM (n ≥ 4).

### SPRR-2 is activated by MIP-related neuropeptides

The *C*. *elegans* ortholog SPRR-2 of the insect MIP receptor is an orphan GPCR, meaning that its ligand(s) is unknown. To identify its ligand(s), we used a calcium (Ca^2+^)-based reporter assay for GPCR activation wherein SPRR-2 is expressed in Chinese Hamster Ovary (CHO) cells (Fig 2A). We challenged these receptor-expressing cells with a synthetic library of ~350 *C*. *elegans* neuropeptides. Only five neuropeptides derived from two predicted protein isoforms of the *nlp*-38 precursor gene activated SPRR-2, whereas they did not activate SPRR-1 or SPRR-3 (S3A and S3B Fig). These peptides (NLP-38-1 to 5) all have a C-terminal glycine residue (G) indicative of post-translational modification into an amide (-NH_2_) group in the mature peptides [32]. In addition, these peptides display the C-terminal sequence W-X_5-8_-Wamide (Fig 2B), which is typical for MIP neuropeptides in other protostomian species [24]. Because of its relationship to protostomian MIP neuropeptide precursors, we refer to the NLP-38 precursor from here onwards as "MIP-1".

We next determined the potency of synthetic MIP-1 peptides to activate SPRR-2. MIP-1-1 to MIP-1-4 showed dose-dependent activation with EC_50_ values in the nanomolar range (7.5 nM– 16 nM), which is in accordance with the low concentrations typical for neuropeptide-GPCR signaling (Fig 2C). The predicted MIP-1-5 peptide also activated SPRR-2 dose-dependently, but with a higher EC_50_ value (486 nM) (Fig 2C).

### MIP-1 neuropeptides are neuronally expressed and required for gustatory plasticity

**Fig 3:**
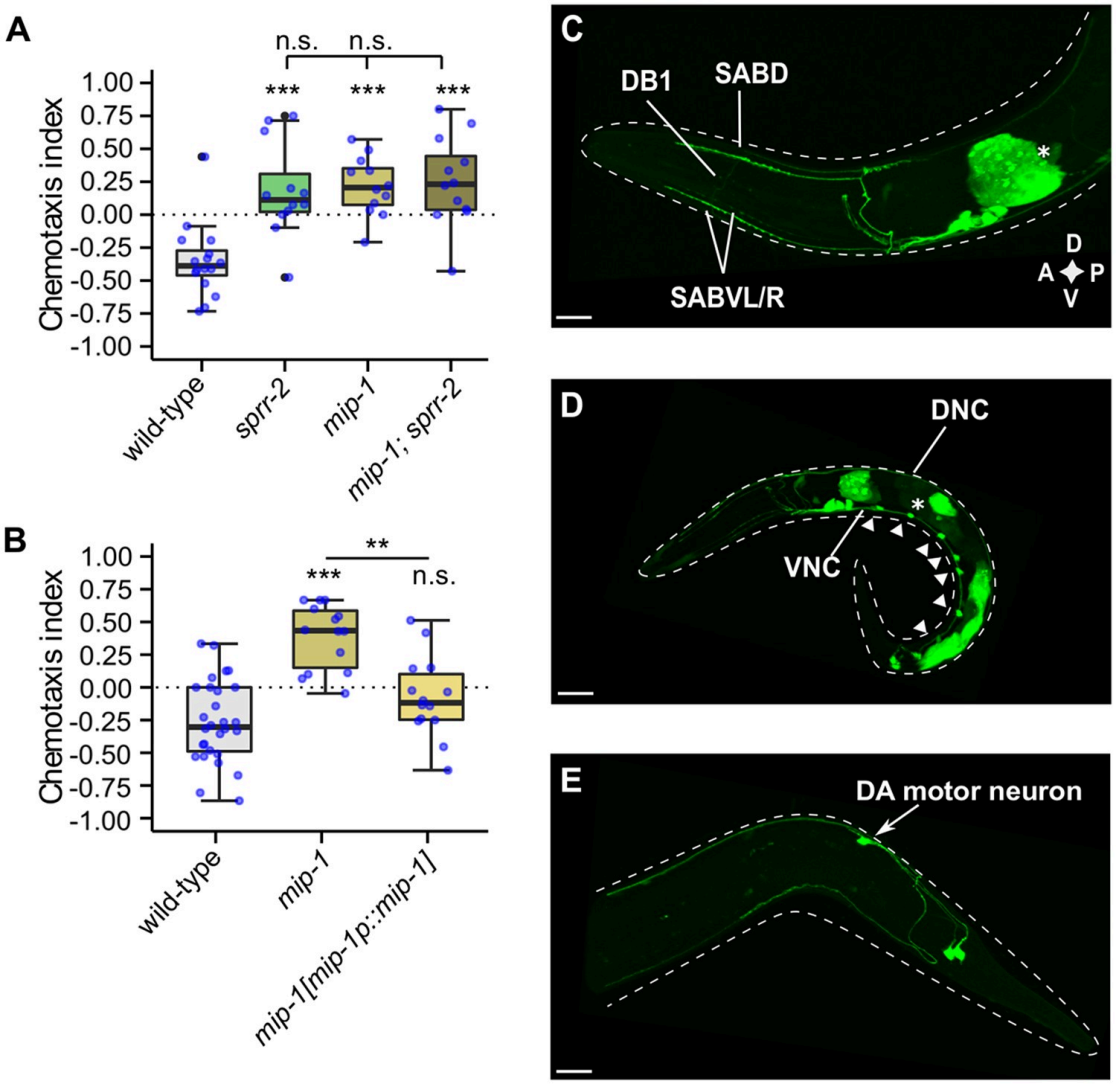
MIP-1 neuropeptides are required for gustatory plasticity. (A-B) Gustatory plasticity of *mip-1* mutant. Individual CIs, blue dots. Outliers, black dots. Boxplots indicate 25^th^ (lower boundary), 50^th^ (line), and 75^th^ (upper boundary) percentiles. Whiskers show minimum and maximum values. Data were analyzed by one-way ANOVA with Tukey post-hoc test (n ≥ 8). *p<0.05; **p<0.005; ***p<0.001; n.s., not significant. (A) *mip-1* mutants are defective in gustatory plasticity. A double mutant of *mip-1* and *sprr-2* has no additive learning defect. (B) The plasticity defect of *mip-1* is rescued by restoring *mip-1* expression under control of its promoter sequence [*mip-1p*::*mip-1*]. (C-E) Labeled confocal Z-stack projections showing expression of a reporter transgene for *mip-1* [*mip-1p*::*mip-1*::*sl2*::*gfp*] in adult (C & E) or larval (D) hermaphrodites. Asterisks mark intestinal fluorescence resulting from the co-injection marker *elt-2p*::*mCherry*. Scale bars represent 15 μm. (C) *mip-1* expression localizes to SABD, SABV and DB-1 motor neurons as well as around 4 additional unidentified neuron pairs in the head. (D) Expression was also found in dorsal and ventral nerve cords (DNC, VNC), and in motor neurons along the VNC (arrowheads). (E) *mip-1* expression in the tail localizes to DA motor neurons, most likely DA8 or DA9, and an additional unidentified neuron pair. D, dorsal; V, ventral; A, anterior; P, posterior.

The specific activation of SPRR-2 by MIP-1 neuropeptides *in vitro* suggests that they are the cognate ligands of this receptor *in vivo*. If this hypothesis is correct, loss-of-function mutants for *mip-1* should display gustatory plasticity defects mimicking those of *sprr-2* mutants. As expected, *mip-1* mutants showed reduced gustatory plasticity compared to wild-type animals and no general defects in locomotion or salt chemotaxis behavior (Fig 3A and S1C–S1E Fig). Moreover, a double mutant of *mip-1* and *sprr-2* did not display an additive plasticity defect as compared to the single mutants, further confirming that MIP-1 signaling through SPRR-2 promotes gustatory associative learning (Fig 3A). The plasticity defect of *mip-1* mutants was rescued by restoring *mip-1* expression (Fig 3B). Hence, the MIP-1 neuropeptide precursor is also required for gustatory plasticity.

**Fig 4:**
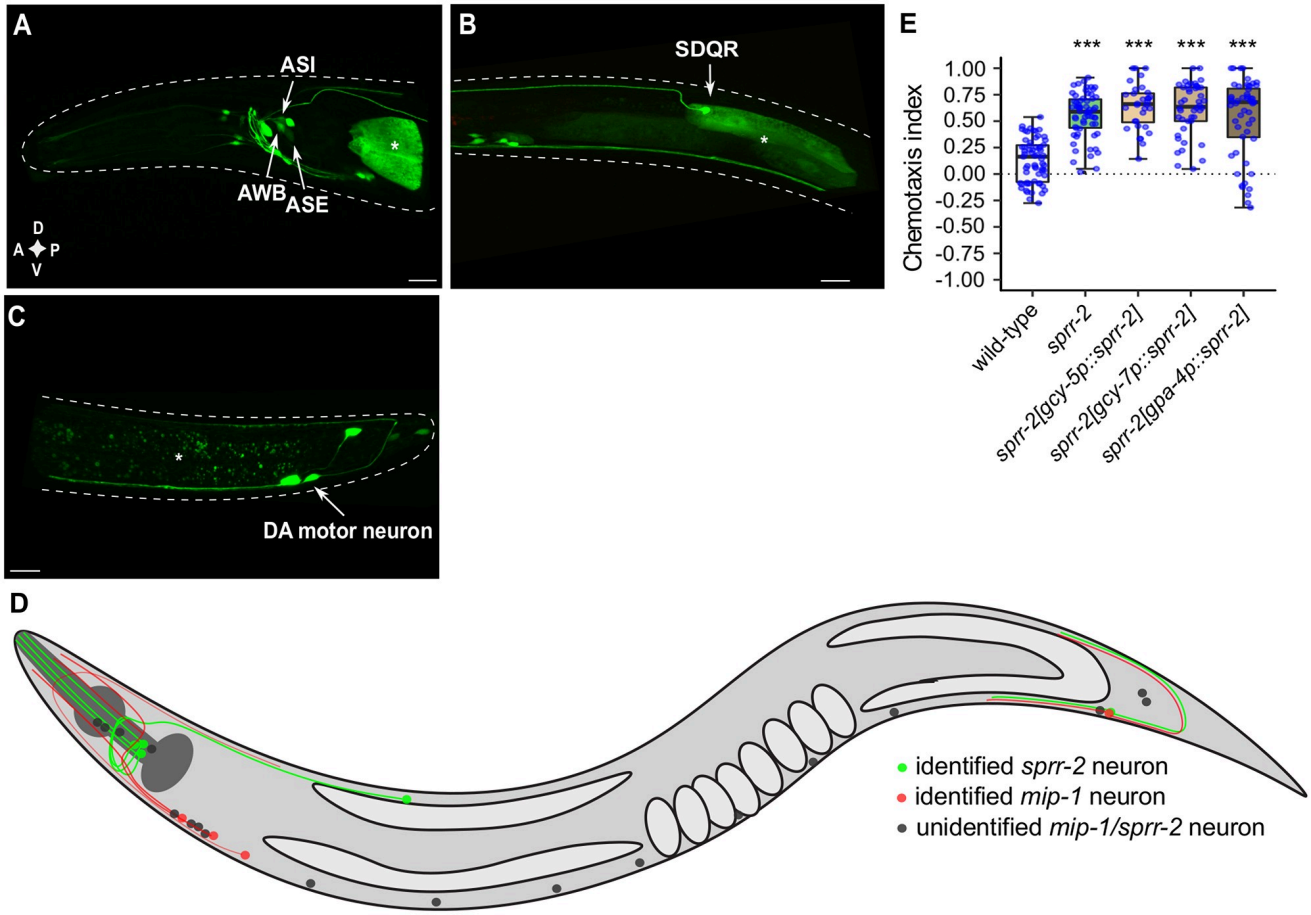
Expression pattern of the MIP receptor gene *sprr-2*. (A-C) Labeled confocal Z-stack projections showing expression of the *sprr-2* fluorescent reporter transgene [*sprr-2p*::*sprr-2*::*sl2*::*gfp*] in adult hermaphrodites. Asterisk marks fluorescence in intestine resulting from the co-injection marker *elt-2p*::*mCherry*. Scale bars, 15 μm. (A) *sprr-2* is expressed in several head neurons (around 10 pairs) of which ASE was identified by crossing with an ASE marker strain OH4165 (S4A Fig). ASI and AWB sensory neurons were identified by DiI staining (S4B Fig). (B) Expression in SDQR along the midbody. (C) *sprr-2* expression in the tail localizes to 3 neurons, one of which is a DA motor neuron, most likely DA8 or DA9. D, dorsal; V, ventral; A, anterior; P, posterior. (D) Schematic representation of the left lateral view of identified *mip-1* (red) and *sprr-*2 neurons and their projections (green) as well as unidentified neurons (gray). (E) Cell-specific expression of *sprr-2* in ASER [*gcy-5p*::*sprr-2*], ASEL [*gcy-7p*::*sprr-2*], or ASI [*gpa-4p*::*sprr-2*] does not rescue the gustatory plasticity defect of NaCl-conditioned *sprr-2* mutant animals. Individual CIs are indicated as blue dots. Boxplots indicate 25^th^ (lower boundary), 50^th^ (line), and 75^th^ (upper boundary) percentiles. Whiskers show minimum and maximum values. Statistical comparisons by one-way ANOVA and Tukey post-hoc test (n ≥ 12). ***p<0.001.

Using tandem mass spectrometry, we identified four of the five predicted neuropeptide sequences of the NLP-38 precursor isoforms (NLP-38-1 to 4), indicating that they are processed and cleaved from their precursor proteins *in vivo* [22,33,34]. We next investigated the spatial expression pattern of the *mip-1* precursor and *sprr-2* genes. Expression of fluorescent reporter constructs for *mip-1* localized to the ventral and dorsal nerve cords, along with several ventral cord motor neurons (Fig 3D, see Methods). Additionally, we observed *mip-1* expression in around 8 neurons in the head and 3 neurons in the tail (Fig 3C–3E). Based on position and unique morphology of their projections, we identified the SAB and DB1 motor neurons in the head, which are involved in forward locomotion. For *sprr-2*, we observed expression in around 10 neuron pairs in the head (Fig 4A), of which the cellular identity was confirmed for the main salt-sensory ASE neurons as well as the ASI and AWB chemosensory neurons. The identity of ASI and AWB neurons was confirmed by diI staining, while ASE expression was validated by co-localization of the GFP signal with an RFP protein expressed from the *ceh-36* promoter, marking ASE and AWC (S4A and S4B Fig). In addition, *sprr-2* expression localized to the SDQ neurons in the midbody (Fig 4B) and was also evident in 3 tail neurons, one of which corresponds to the DA8 or DA9 motor neuron (Fig 4C). A diagram showing *mip-1* and *sprr-2* expressing neurons is provided in Fig 4D and shows that there appears to be no or limited overlap between *mip-1* and *sprr-2* expressing neurons.

Because ASE and ASI neurons are part of the gustatory plasticity circuit [35], we investigated whether cell-specific expression of *sprr-2* in ASI, ASEL or ASER neurons is sufficient to rescue the plasticity defect. Our findings imply that cell-specific expression of *sprr-2* in any of these neurons individually is insufficient to restore aversive learning (Fig 4E).

### MIP-1/SPRR-2 signaling promotes salt avoidance learning, which displays hallmarks of long-term memory

**Fig 5:**
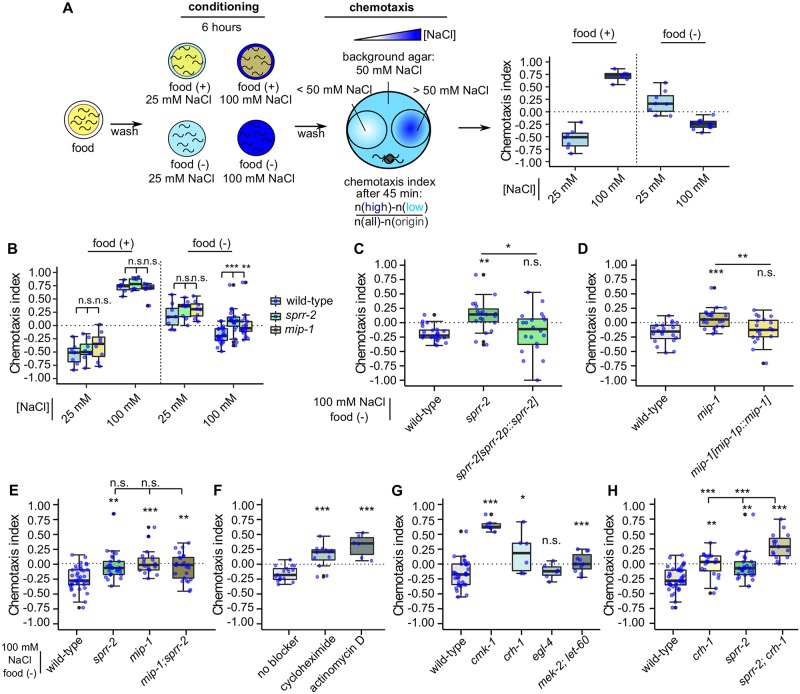
MIP-signaling mediates salt avoidance learning that shows hallmarks of long-term memory. (A) Overview of taste associative learning assays. Synchronized young adult *C*. *elegans* are conditioned for six hours on NaCl-containing plates with or without bacterial food. Salt chemotaxis behavior of worms is then tested on NaCl concentration gradients. The CI is calculated from the number of worms that migrated toward the lower and upper parts of the gradient [8–10]. Worms chemotax to the salt concentrations at which they were fed, but avoid salt concentrations at which they were starved. (B) Mutants of *mip-1* and *sprr-2* chemotax to the salt concentrations at which they were fed and do not show any significant salt avoidance learning defects compared to wild-type animals when conditioned at 25 mM NaCl in the absence of food, but do show significantly impaired salt avoidance learning at 100 mM NaCl. (C) Expression of *sprr-2* under its promoter sequence [*sprr-2p*::*sprr-2*] rescues the salt avoidance learning defect of *sprr-2* mutants. (D) Expression of *mip-1* under control of its endogenous promoter sequence [*mip-1p*::*mip-1*] rescues the salt avoidance learning defect of *mip-1* mutants. (E) A *mip-1; sprr-2* double mutant has no additive learning defect. (F) Blocking translation (cycloheximide) or transcription (actinomycin D) during conditioning impairs salt avoidance learning in wild-type animals. (G) Salt avoidance learning of mutants for transcription factors and regulators linked to learning and memory. (H) A *sprr-2; crh-1* double mutant displays a statistically significant additive learning defect as compared to the single mutants. For (B-H), boxplots indicate 25^th^ (lower boundary), 50^th^ (line), and 75^th^ (upper boundary) percentiles. Whiskers show minimum and maximum values. Individual CIs, blue dots. Outliers, black dots. Statistical comparisons by one-way ANOVA and Tukey post-hoc test (n ≥ 6). *p<0.05; **p<0.005; ***p<0.001; n.s., not significant.

We next explored the behavior of *sprr-2* mutants in a second established type of gustatory associative learning that depends on food availability and external salt concentration, generally referred to as taste associative learning [8,10,36]. Previous work showed that wild-type worms are attracted to the NaCl concentration at which they were fed [8]. In contrast, they are averted by NaCl concentrations at which they were starved, a behavior that is called salt avoidance learning [8,9] (Fig 5A, see Methods). To test taste associative learning of *sprr-2* mutants, we fed or starved animals, respectively, for six hours on conditioning plates with 25 mM NaCl (low salt) or 100 mM NaCl (high salt). We found that *sprr-2* mutants fail to avoid the high salt concentration experienced during starvation: in contrast to wild-type animals, *sprr-2* mutants did not migrate to low salt concentrations after exposure to 100 mM NaCl in the absence of food (Fig 5B). This salt avoidance learning defect could be rescued by reintroducing wild-type copies of *sprr-2* under control of its endogenous promoter (Fig 5C). Similar to *sprr-2* mutants, *mip-1* mutants showed decreased migration to low salt concentrations after exposure to 100 mM NaCl in the absence of food (Fig 5B). This defect could be rescued by reintroducing *mip-1* under its endogenous promoter in the mutant background (Fig 5D). Furthermore, a *mip-1; sprr-2* double mutant did not display an additive defect as compared to the single mutants (Fig 5E), indicating that MIP-1 signaling via SPRR-2 modulates salt avoidance learning towards high salt.

Because salt avoidance learning relies on a conditioning period of several hours, we questioned whether this type of learning generates long-term memory and by extension whether MIP signaling is involved in the modulation of both short- and long-term gustatory learning. We first tested whether salt avoidance learning relies on protein synthesis by *de novo* transcription and translation, which are two hallmarks of long-term memory. We thereto blocked transcription or translation during the conditioning phase by respectively administering actinomycin D or cycloheximide to the conditioning plates. Both actinomycin D and cycloheximide treatment significantly weakened the avoidance of high salt concentrations experienced during starvation (Fig 5F, see Methods), suggesting that this type of memory is indeed transcription- and translation-dependent. Administration of either of these blockers during conditioning with 100 mM NaCl in the presence of food did not affect wild-type learning behavior (S5A Fig). Hence, potential side effects of the chemical treatments are unlikely to underlie the learning defect observed in salt avoidance learning.

We further explored the transcription-dependency of salt avoidance learning by investigating whether it depends on the activation of cAMP response element binding protein (CREB), a transcription factor crucial for the formation of long-term memory from *Aplysia* to humans [1,37–39]. To this end, we tested null mutants of the *C*. *elegans* CREB ortholog, *crh-1*, in salt avoidance learning together with mutants of various kinases that can regulate the phosphorylation status of CREB, including Ca^2+/^calmodulin-dependent kinase mutants *cmk-1*, cyclic GMP-dependent protein kinase mutants *egl-4*, and a double mutant *mek-2;let-60* disrupting Ras/mitogen activated protein kinase (MAPK) signaling [39–43]. While salt avoidance learning was unaffected in *egl-4* mutants, mutants of *cmk-1* and *crh-1* were still attracted to high NaCl concentrations after pairing salt with starvation (Fig 5G). Double mutants with disrupted MAPK signaling (*mek-2;let-60)* showed no preference to high or low salt concentrations. Although these factors may serve broader functions in the nervous system, these findings suggest that salt avoidance learning involves activation of the CREB pathway. Taken together, our results indicate that this type of learning depends on *de novo* transcription and translation, which is a key hallmark of long-term memory.

We next questioned whether MIP-1/SPRR-2 signaling acts together with CREB signaling to mediate salt avoidance learning. Mutants lacking the CREB homolog *crh-1* displayed normal salt chemotaxis behavior for NaCl concentrations under study after mock-conditioning (S5B Fig), but showed a clear defect in salt avoidance learning after long-term conditioning with high salt in absence of food (Fig 5H). We next explored the behavior of a *crh-1;sprr-2* double mutant in the salt avoidance learning paradigm. Interestingly, a double mutant of *crh-1* and *sprr-2* displayed a significantly stronger defect in salt avoidance learning compared to the single mutants, which suggests that *sprr-2* modulates salt avoidance learning in parallel with *crh-1* (Fig 5H).

### MIP signaling through SPRR-2 modulates salt avoidance learning in parallel with the insulin pathway

**Fig 6:**
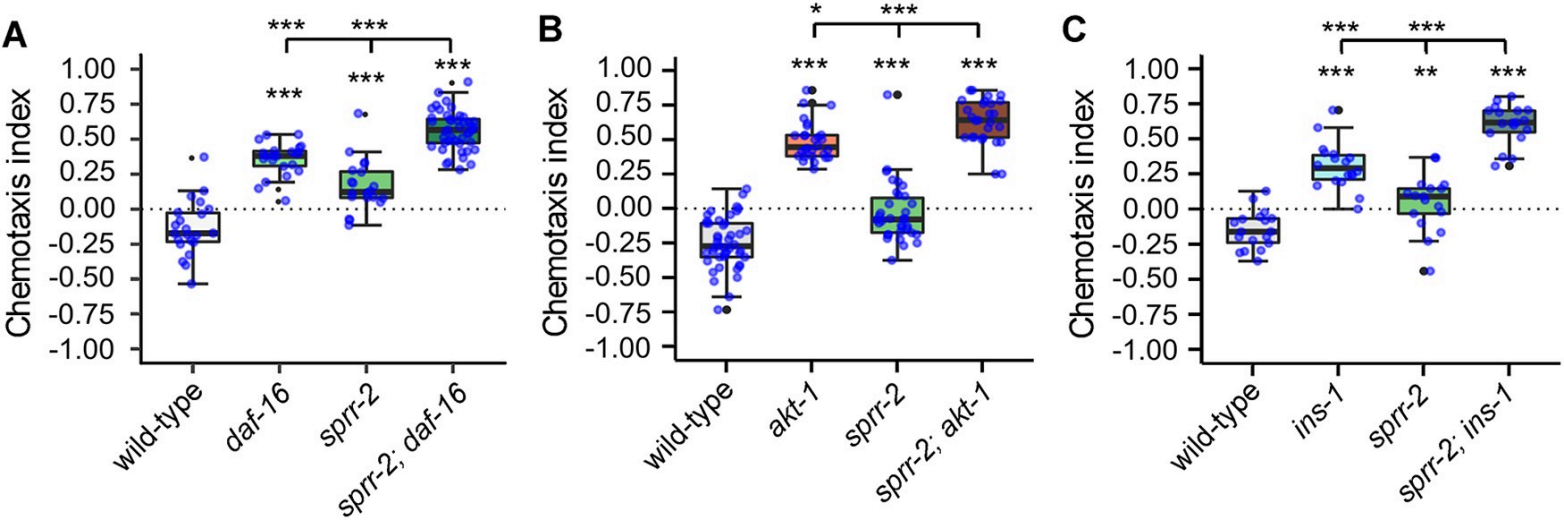
MIP-signaling mediates salt avoidance learning in parallel with the insulin pathway. (A) *sprr-2* does not genetically interact with *daf-16* to regulate salt avoidance learning as *sprr-2; daf-16* double mutants show further impaired learning as compared to the single mutants. (B) *sprr-2; akt-1* and (C) *sprr-2; ins-1* double mutants also show a significantly enhanced learning defect as compared to their respective single mutants. Individual CIs are plotted as blue dots. Boxplots indicate 25^th^ (lower boundary), 50^th^ (line), and 75^th^ (upper boundary) percentiles. Whiskers show the minimum and maximum values. Outliers are indicated as black dots. Data were analyzed by one-way ANOVA and Tukey post-hoc test (n ≥ 17). *p<0.05, **p<0.005, ***p<0.001.

Previous studies established that mutants of the insulin/PI3K pathway fail to avoid NaCl concentrations associated with starvation, although they show normal attraction to NaCl concentrations experienced during feeding [8,10,44]. We therefore investigated the genetic interaction between *sprr-2* and components of the insulin pathway. We generated double loss-off-function mutants of *sprr-2* with *daf-16*, *akt-1* and *ins-1*. After conditioning worms with 100 mM NaCl without food, *sprr-2; daf-16* double mutants showed a significantly stronger defect in salt avoidance learning as compared to the single mutants (Fig 6A), indicating that *sprr-2* mediates salt avoidance learning in parallel with *daf-16*. We obtained similar results for *akt-1* and *ins-1* since *sprr-2; akt-1* and *sprr-2; ins-1* double mutants both displayed significantly enhanced defects as compared to their respective single mutants (Fig 6B and 6C). Taken together, these results suggest that MIP signaling through SPRR-2 promotes salt avoidance learning in parallel with the insulin pathway.

## Discussion

### MIP signaling is a novel regulator of short-term gustatory plasticity

In this study, we show that MIP signaling modulates gustatory plasticity, a type of short-term aversive learning. *C*. *elegans* has three predicted MIP receptor orthologs: SPRR-1, SPRR-2, and SPRR-3 (*s*ex *p*eptide *r*eceptor *r*elated), belonging to the rhodopsin class of GPCRs and originally named after their sequence similarity with the *Drosophila* sex peptide receptor (SPR) [24,25,27,28,45]. This insect MIP receptor was initially named SPR because it is activated *in vitro* by the *Drosophila*-specific sex peptide that is secreted from the male accessory glands [29]. Later, in 2010, two groups independently demonstrated that this receptor is in fact the cognate receptor for MIPs [24,25]. Not the *Drosophila*-specific sex peptide, but the evolutionarily highly conserved MIPs are the likely ancestral ligands of the SPR receptors, which we therefore propose to rename as MIP receptors.

We show that neuropeptides encoded by the NLP-38 neuropeptide precursor, referred to as MIP-1, activate the MIP receptor SPRR-2 in *vitro*. The MIP-1 neuropeptide precursor in *C*. *elegans* encodes five peptides that all exhibit the characteristic MIP C-terminal W-X_5-8_-Wamide motif. This coincides with MIP precursors in insects, which typically produce five to seven mature MIPs that are generally short peptides, containing 9 to 12 amino acids [18,24].

In addition to activating SPRR-2 *in vitro* we provide evidence that MIP-1 neuropeptides act through SPRR-2 activation in gustatory plasticity *in vivo*. Whereas wild-type worms learn to avoid salt after exposure to it in the absence of food, null mutants of *mip-1* and *sprr-2* show significantly reduced NaCl aversion after conditioning. These learning defects could be rescued by re-introducing *mip-1* or *sprr-2* under control of their endogenous promoters in the respective mutants. Moreover, a *mip-1; sprr-2* double mutant showed no additive learning defect compared to the single mutants, suggesting that MIP-1 neuropeptides act through SPRR-2 *in vivo*. Given that we showed that the *C*. *elegans* receptor SPRR-2 is activated by MIP-1 peptides *in vivo*, whereas sex peptides appear to be *Drosophila*-specific, we propose to rename SPRR-2 as MIPR-1.

We localized *sprr-2* expression to the sensory ASE and ASI neurons, amongst other cells. These head neurons are part of the gustatory plasticity circuit [35]. ASE neurons are the major sensors for salts and water-soluble attractants, primarily mediating sensory responses to attractive NaCl concentrations [46,47] and consist of two morphologically symmetric neurons on the left (ASEL) and the right (ASER) [48]. Both ASEL and ASER regulate gustatory learning, but use distinct molecular mechanisms [49]. Insulin signaling modulates gustatory learning in ASER, whereas nematocin signaling routes through ASEL [10,11,49–51]. It has been proposed that upon prolonged NaCl exposure during starvation, ASE neurons sensitize ASI neurons, amongst others, thereby resulting in avoidance of otherwise attractive NaCl concentrations and as such contributing to gustatory learning [35]. Although ASE and ASI neurons are likely candidates of the cellular circuit underlying *mip-1/sprr-2*-mediated aversive gustatory learning, we showed that cell-specific expression of *sprr-2* in either of these cells alone is insufficient to rescue gustatory plasticity. One explanation could be that *sprr-2* expression is required in multiple cells to mediate gustatory plasticity.

### MIP signaling promotes salt avoidance learning, reminiscent of long-term memory, in parallel with the insulin and CREB pathway

In addition to gustatory plasticity, we explored the behavior of *sprr-2* mutants in a second type of gustatory associative learning that depends on food availability and salt concentration [8,10,36]. We evaluated the behavior of *sprr-2* mutants on a salt chemotaxis gradient under four salt conditioning regimes: pairing high and low salt concentrations with food or with starvation. Wild-type animals are attracted to the NaCl concentration at which they were previously fed, whereas they avoid NaCl concentrations at which they were starved [8–10]. We found that *sprr-2* mutants fail to avoid high salt concentrations (100 mM NaCl) after starvation. This salt avoidance learning defect could be rescued by reintroducing *sprr-2* under control of its endogenous promoter. Similarly to the learning defect of *sprr-2* mutants, *mip-1* deletion mutants failed to avoid high salt concentrations after conditioning with 100 mM NaCl in the absence of food, a defect that could also be rescued by reintroducing *mip-1* under control of its endogenous promoter. Moreover, a *mip-1;sprr-2* double mutant did not display an additive defect as compared to the single mutants, indicating that MIP-1 signaling through SPRR-2 modulates salt avoidance learning in response towards high NaCl levels.

Our analysis of salt avoidance learning suggests that it displays several hallmarks of long-term memory. First, we found that blocking translation or transcription by cycloheximide or actionomycin D treatment during conditioning disrupted learned salt aversion, suggesting that it requires *de novo* transcription and translation. The requirement of transcription and translation has been found crucial for the formation of long-term memory in a variety of model systems, but not short-term memory [1]. Second, we found that mutants of *C*. *elegans* orthologs of CREB (*crh-1*), calmodulin kinase (*cmk-1*) and the Ras-MAPK pathway (*mek-2; let-60*) all display defects in salt avoidance learning. This finding suggests that salt avoidance learning requires the activation of these signaling pathways, although we cannot rule out that they may indirectly affect learning through other functions in the nervous system. CREB is a transcription factor crucial for the formation of long-term memory from *Aplysia* to humans, as well as olfactory long-term memory in *C*. *elegans* [1,3,5,38,39,52–54]. During long-term memory formation, CREB activity is regulated by its phosphorylation status which is influenced by various kinases such as Ca^2+/^calmodulin-dependent kinases and proteins of the Ras/MAPK pathway [39,40]. *C*. *elegans* mutants of these kinases, including *cmk-1* as well as mutants of the MAPK-pathway are also known to be defective in the formation of olfactory long-term memory, further suggesting that MIP-1/SPRR-2 signaling promotes aversive gustatory learning reminiscent of long-term memory in addition to short-term gustatory aversive learning [42,43].

Since MIP-1/SPRR-2 as well as CREB signaling appears to modulate salt avoidance learning towards high salt, we investigated whether MIP signaling through SPRR-2 directly routes through CREB. Interestingly, our genetic analysis indicated that *sprr-2* and *crh-1* do not act in the same genetic pathway, because a *crh-1;sprr-2* double mutant displayed an additive learning defect compared to the single mutants. We next examined whether MIP-1 signaling through SPRR-2 genetically interacts with the insulin/PI3K pathway, as previous studies showed that salt avoidance learning is regulated by this pathway [10,44,49]. One downstream component of the insulin pathway is the FOXO transcription factor DAF-16, which we found to act in parallel with the *sprr-2* pathway [55]. Previous reports have suggested that *daf-16* is not the sole downstream target of insulin signaling in gustatory associative learning [10]. We therefore also investigated the genetic interaction between *sprr-2* and two additional components of the insulin pathway: the insulin-like peptide INS-1 and the protein kinase B AKT-1 [55,56]. In accordance with the results we obtained for *daf-16*, double mutants of *sprr-2;akt-1* and *sprr-2;ins-1* learned significantly worse than the respective single mutants. Hence, MIP-1 signaling through SPRR-2 appears to regulate salt avoidance learning in parallel with the insulin pathway.

The question remains how MIP-1/SPRR-2 signaling modulates salt avoidance learning towards high salt. In addition to *crh-1* we identified *cmk-1* and mutants of the Ras-MAPK pathway to be defective in salt avoidance learning. Hence, MIP-1/SPRR-2 signaling may affect either of these kinase cascades in parallel with *crh-1* signaling. Alternatively, *sprr-2* may interact with the evolutionarily conserved mammalian target of rapamycin (mTOR) pathway. This pathway has recently been shown to be implicated in salt avoidance learning and plays a major role in synaptic plasticity and memory due to its critical role in protein synthesis [36,57].

### Homology between *C*. *elegans* and mammalian MIP systems

So far, MIP receptors and neuropeptides have only been studied in protostomian animals and until recently, MIP signaling was assumed to be a typical invertebrate evolutionary invention. Whereas bioinformatic analysis employing a small set of closely related receptors did not identify any apparent vertebrate orthologs [29], more recent bioinformatic clustering analyses investigating the long range evolution of neuropeptide systems across metazoans revealed that orthologs of MIP receptors also occur in animal genomes of the deuterostomian lineage, including humans, fish, amphibians, reptiles, birds, mammals, and the more ancient chordates such as *Branchiostoma* [27,28]. The human receptor orthologs of MIP receptors are GPR139 and GPR142, two orphan GPCRs for which neither the endogenous ligand nor the function has been elucidated. GPR139 is predominantly expressed in specific areas of the human and mouse CNS, including the amygdala and hippocampus, whereas GPR142 displays a more ubiquitous expression both in the CNS and in various peripheral glands and organs [31,58,59]. Interestingly, it has been shown, for example, that avoidance learning in humans correlates with activation of amygdala [31,58,59]

MIP neuropeptide sequences have as yet not been identified in humans, nor in any other deuterostomian species [27,28]. However, it has been suggested that the cognate ligand(s) for GPR139 and GPR142 is present in brain extracts and is possibly a small peptide [31]. More recently, L-Trp (W) and L-Phe (F) have been suggested as candidate ligands from *in vitro* studies with synthetic compounds [60]. MIPs belong to the W-amide neuropeptide superfamily, which consists of (G)LWamides and many other Wamide-type peptides that are also found in the ancient eumetazoan cnidarian phylum [18]. All Wamide neuropeptide family members share an amidated Trp (W) residue preceded by a small aliphatic residue. Given the agonist activity of L-Trp (W) and analogs on the human MIP receptor orthologs, it is conceivable to predict that the deuterostomian MIP receptors, GPR139 and/or GPR142, are activated by such Wamide-type peptides.

Our findings expand our current knowledge on the neuropeptidergic modulation of learning and memory, characterizing a system modulating both short-term memory and memory dependent on *de novo* protein synthesis. As MIP receptor orthologs are suggested to be conserved from worms to humans, the MIP signaling system most likely dates back prior to the split of protostomian and deuterostomian phyla, more than 700 million years ago, and may, because of its vital importance, have been evolutionarily conserved since. This study lays a foundation for the characterization of the orphan human MIP receptors, which are potential targets for cognitive disorder treatments, and for further understanding on how aversive events in life become anchored in memory. With the recent increased interest in avoidance learning from psychology literature [30], this study contributes to the integration of insights from neuroscience in psychological theories of avoidance learning.

## Materials and methods

### *C*. *elegans* strains and culture

All *C*. *elegans* strains were maintained at 20°C on nematode growth medium (NGM) plates seeded with *E*. *coli* OP50 as a food source, unless stated otherwise. All *C*. *elegans* behavioral experiments were done using hermaphrodite young adults and were performed in a climate controlled room at 20°C and 40% relative humidity. For gustatory plasticity experiments, synchronized worms were grown for one generation at 25°C until they reached young adulthood. All transgenic strains used for localization were made by microinjection of plasmid DNA into N2 hermaphrodites. Adult hermaphrodite worms were used for DiI staining and confocal imaging. Rescue strains were made by microinjection of plasmid DNA/PCR products into the respective null mutants. Strains used in this study and their corresponding figures are listed in S1 Table. All animal experiments were performed in accordance with the governmental and institutional guidelines.

### Molecular biology

For heterologous expression of SPRR-2 in CHO cells, *sprr-2* cDNA was cloned into a pcDNA3.1/V5-His-TOPO vector [11,20,61]. cDNA sequences were PCR amplified from mixed-stage wild-type *C*. *elegans* template. GFP reporter and rescue constructs were generated by using a modified pSM vector carrying a GFP reporter sequence preceded by an SL2 trans-splicing sequence (kindly provided by C. Bargmann, Rockefeller University, New York, NY). The pSM vector was linearized by KpnI digestion after which *sprr-2* cDNA or gDNA, or *mip-1* gDNA was cloned in by Gibson Assembly (NEB). Putative promoter sequences (*sprr-2p*: 1.9kb and *mip-1p*: 2 kb) were inserted into the vector by Gibson Assembly after linearization by BamHI digestion. For ASER-specific rescue of *sprr-2*, the *gcy-5* putative promoter sequence (~3kb) was inserted into the pSM vector containing the *sprr-2* cDNA by Gibson assembly. ASI- and ASEL-specific rescue constructs were made by fusing the *gpa-4p* (~2.5 kb) and *gcy-7p* (~1.3kb) promoters to the *sprr-2* cDNA PCR fragment [62]. S2 Table provides an overview of plasmids generated in this study and the primer sequences used for cloning.

### Transgenesis and expression pattern analysis

Germline transformations were carried out by injecting constructs into the syncytial gonad of young adult worms at concentrations ranging from 10 to 50 ng/μL with 50 ng/μL of co-injection marker and 0.5 μL of a 1-kb DNA ladder (0.5 μg/μL) (Thermo Scientific) as carrier DNA. *elt-2p*::*mCherry*, *unc-122p*::*dsRED*, or *unc-122p*::*GFP* was used as a co-injection marker.

Expression patterns of GFP reporter transgenes were visualized by an Olympus Fluoview FV1000 (IX81) confocal microscope and confocal Z-stack projections were analyzed with Imaris 7.2 (Bitplane) software. For imaging, hermaphrodite animals were mounted on 2% agarose pads with 500 mM sodium azide (Sigma-Aldrich) in M9 buffer. Expression patterns were confirmed in at least two independent transgenic strains. For *sprr-2*, reporter constructs containing the gene’s genomic DNA or cDNA showed similar expression patterns. Expression in AWB and ASI neurons was confirmed by DiI (1,1'-Dioctadecyl-3,3,3',3'-Tetramethylindocarbocyanine Perchlorate, Invitrogen) staining [63]. ASE expression was confirmed by crossing with marker strain OH4165, which marks ASE and AWC neurons. Cells expressing *mip-1* were identified based on their position and morphology.

### Gustatory plasticity

Gustatory plasticity assays were performed as described previously [11–13,64] in a climate controlled room at 20°C and 40% relative humidity (Fig 1A). Synchronized hermaphrodites were grown at 25°C until they reached young adulthood. Worms were washed three times for 5 minutes with chemotaxis (CTX) buffer (5 mM KH_2_PO_4_/K_2_HPO_4_ pH 6.6, 1 mM MgSO_4_, and 1 mM CaCl_2_) without salt (mock-trained) or supplemented with salt (100 mM NaCl, conditioned). After 15 minutes conditioning, the buffer was removed and chemotaxis towards NaCl was tested. NaCl chemotaxis was assayed on quadrant plates filled with buffered agar (2% agar, 5 mM KH_2_PO_4_/K_2_HPO_4_ pH 6.6, M MgSO_4_, 1 mM CaCl_2_) with or without salt (25 mM NaCl). Between 30 and 150 worms were placed on the centre of the chemotaxis plate. After 10 minutes, worms on each quadrant were counted and the chemotaxis index (CI) was calculated as: CI = N(A)—N(C) / N(A) + N(C). N(A) represents the number of worms within quadrants supplemented with NaCl, whereas N(C) is the number of worms on quadrants without NaCl.

Salt sensing experiments (S1E and S5B Figs) were performed as described for gustatory plasticity with CTX buffer without salt (mock-trained). After 15 minutes mock-conditioning, the buffer was removed and chemotaxis towards different NaCl concentrations was tested. NaCl chemotaxis was assayed on quadrant plates filled with buffered agar (2% agar, 5 mM KH_2_PO_4_/K_2_HPO_4_ pH 6.6, M MgSO_4_, 1 mM CaCl_2_) with or without salt (0.1 mM, 10 mM, 100 mM, 200 mM, or 500 mM NaCl).

For pairing of NaCl with food, conditioning was carried out on plates (S2A Fig). Well-fed synchronized young adults were collected in CTX buffer with 100 mM NaCl (conditioned) and then transferred to conditioning plates (2% agar, 100 mM NaCl, 5 mM KH_2_PO_4_/K_2_HPO_4_ pH 6.6, M MgSO_4_, and 1 mM CaCl_2_) that were prepared and seeded with 200 μL of a 0.5 g/mL *E*. *coli* OP50 solution (in MQ) on the day of the assay. After 30 minutes conditioning, worms were washed off the plates with CTX buffer + 100 mM NaCl and chemotaxis towards NaCl was assessed in the quadrant plate assay.

Training protocols and corresponding figures are summarized in S3 Table.

### Taste associative learning assays

Taste associative learning assays were performed as previously described (Fig 5A) [8].

Worms were washed off the cultivation plates with chemotaxis buffer (25 mM KH_2_PO_4_/K_2_HPO_4_ pH 6.6, 1 mM MgSO_4_, 1 mM CaCl_2_, 50 mM NaCl) and transferred to unseeded (salt avoidance learning) or seeded NGM conditioning plates with 100 mM NaCl. After 6 hours, animals were washed off and rinsed once with chemotaxis buffer before testing chemotaxis towards NaCl on salt concentration gradients. Salt concentration gradients consisted of a thin layer of 50 mM NaCl 2% agar solution in chemotaxis buffer (background agar, 10 mL was poured into 8.5 cm diameter plates). Additionally, 2% agar in chemotaxis buffer solutions supplemented without 0 mM (lower side) or with 150 mM NaCl (higher side) were prepared by pouring 10 mL agar solution into 35 mm diameter plates. Cylindrical blocks with the diameter of a 15 mL falcon tube were excised from either solution and applied on the background agar 3 cm off centre in opposite directions. Salt concentration gradients were subsequently placed at 20°C for 18 hours before removing the agar chunks prior to use. 30 to 150 worms were placed at the origin and allowed to move freely for 45 minutes after which the CI was calculated as N(higher)—N(lower) / N(all)—N(origin) where N(higher) or N(lower) is the number of animals within a 2-cm radius from the centre of the blocks. Animals within a 1-cm radius from the origin were excluded from the assay. A CI of +1.0 indicates a preference for higher salt concentrations and a CI of -1.0 indicates a complete preference of low salt concentrations. An index of zero can either represent preference for the background concentration, equal distribution to both sides or a random distribution.

Drug treatment for blocking translation with cycloheximide and transcription with actinomycin D was performed in accordance with previous studies [52,65]. Blockers were added to the conditioning plates at a final concentration of 300 μg/mL for cycloheximide (Sigma-Aldrich) and 50 μg/mL for actinomycin D (Sigma-Aldrich). Actinomycin D conditioning plates were prepared and stored in the dark.

Training protocols and associated figures are summarized in S3 Table.

### Locomotion assays

For on-food locomotion assays, around 10 well-fed young adults were transferred from their culture plate to a freshly seeded NGM plate after which they were immediately imaged by an in-house tracking platform consisting of a Rosco 12”x12” LitePad and NET GP11004M cameras fitted with LM16JC10M KOWA lenses. Image acquisition was performed with StreamPix 6 multicamera software for 10 minutes at 2 fps, after which worms were tracked using custom particle-tracking MATLAB code. For off-food locomotion, around 5–10 well-fed worms were picked to an unseeded NGM plate and then within 3 minutes transferred to a second unseeded NGM plate used for imaging, making sure that there was no residual OP50 present on the plates.

### Peptides

A peptide library of 344 synthetic *C*. *elegans* peptides was composed based on *in silico* predictions and in-house peptidomics data [22,33], and was custom-synthesized by Thermo Scientific and GL Biochem Ltd. For dose-response measurements, MIP-1 peptides were purified by reversed phase high performance liquid chromatography on a Symmetry-C18 column (4.6 x 250 mm HPLC cartridge with pore size of 5 μM) and quantified with the bicinchoninic acid (BCA) protein assay. Peptide masses were verified by (matrix assisted laser desorption/ionisation time-of-flight analyzer) MALDI-TOF mass spectrometry on a Reflex IV instrument (Bruker Daltonic).

### In vitro GPCR activation assay

The GPCR activation assay was performed as previously described [11,20,61]. Briefly, CHO cells stably expressing the luminescent Ca^2+^ indicator aequorin and the promiscuous G_α16_ protein (CHO-WTA11 cells, PerkinElmer) were transiently transfected with *sprr-2*/pcDNA3.1, *sprr-1*/pcDNA3.1, *sprr-3*/pcDNA3.1, or empty pcDNA3.1 vector. The G_α16_ protein couples to most agonist-induced GPCRs and directs signaling to the Ca^2+^ pathway regardless of their endogenous G-protein coupling, which allows monitoring GPCR activation by measuring intracellular Ca^2+^ levels [66,67]. Cells were transfected with Lipofectamine LTX and Plus reagent (Invitrogen) at 60–80% confluency and grown overnight at 37 °C. After 24 hours, they were shifted to 28°C overnight. On the day of the assay, transfected cells were collected in bovine serum albumin (BSA) medium (DMEM/F12 without phenol red with L-glutamine and 15 mM HEPES, Gibco, supplemented with 0.1% BSA), at a density of 5 x 10^6 cells per mL, and loaded with 5 μM coelenterazine h (Invitrogen) for 4 hours at room temperature. Compound plates containing synthetic peptides in DMEM/BSA were placed in the Mithras LB940 luminometer (Berthold Technologies). After loading, the transfected cells were added at a density of 25,000 cells/well, and luminescence was measured for 30 s at a wavelength of 469 nm. After 30 s, 0.1% triton X-100 (Merck) was added to lyse the cells, resulting in a maximal Ca^2+^ response that was measured for 10 s. To constitute concentration-response curves of MIP-1 peptides, peptide concentrations ranging from 0.1 nM to 100 μM were tested in duplicate or triplicate on at least two independent days.

### Quantification and statistical analysis

All behavioral experiments were performed at least in duplicate on at least two independent days. Total size number is indicated in figure legends by N. Statistical Significance was determined when p < 0.05 as indicated in the figure legends. For gustatory plasticity and taste associative learning assays, data was statistically analyzed by making use of R (R-3.4.0.–The R project) and R studio (R studio) software using one-way ANOVA and Tukey post-hoc tests for multiple comparisons. GraphPad Prism 5 software (GraphPad) was used to perform Two-way ANOVA and Dunnett’s multiple comparisons test to analyze salt chemotaxis behavior to increasing NaCl concentrations (S1 Fig). For the in vitro GPCR activation assay, concentration-response curves were fitted using GraphPad Prism 5 (nonlinear regression analysis with a sigmoidal concentration-response equation). Locomotion tracking was analyzed using custom particle-tracking MATLAB code (Matlab R2016a—Mathworks). Differences in average worm speed over the 10 minute interval were analyzed by one-way ANOVA and Tukey post-hoc for multiple comparisons and are specified in the figure legends.

## Supporting information

S1 File Source data for Fig 1 and S5 Fig. (XLSX)

S1 Fig (A-D) Average speeds of individual (n ≥ 20) worms off food (A & C) or on an OP50 bacterial lawn (B & D) are scattered as blue dots. Boxplots indicate 25th (lower boundary), 50th (line), and 75th (upper boundary) percentiles. Whiskers show the minimum and maximum values. Outliers are indicated as black dots. Data were analyzed by one-way ANOVA with Tukey post-hoc test. The average speed of sprr-2 and mip-1 mutants is not significantly different (p>0.05) from that of wild-type animals. (E) Mock-conditioned mip-1 and sprr-2 mutants show normal salt chemotaxis behavior to increasing NaCl concentrations. Two-way ANOVA statistical analysis did not reveal any differences in NaCl chemotaxis behavior of mip-1 and sprr-2 mutants as compared to wild-type animals. Mean chemotaxis indices with SD are plotted for wild-type animals and mip-1 and sprr-2 mutants for NaCl concentrations ranging from 0.1 to 500 mM.
(TIF)

S2 Fig (A) NaCl chemotaxis behavior after conditioning salt with food. Synchronized 1-day adult C. elegans were conditioned on NaCl-containing plates in the presence of bacterial food. Chemotaxis behavior to NaCl was then tested on a quadrant plate. The CI is calculated from the number of worms that are present on quadrants with (A) or without (C) NaCl after 10 minutes. (B) NaCl chemotaxis behavior of sprr-2 mutants conditioned with salt in the presence of food is not significantly different (p>0.05) from the behavior of wild-type animals. Data were analyzed by one-way ANOVA with Tukey post-hoc test (n ≥ 10). Boxplots indicate 25th (lower boundary), 50th (line), and 75th (upper boundary) percentiles. Whiskers show the minimum and maximum values. Outliers are indicated as black dots. Individual CIs are plotted as blue dots.
(TIF)

S3 Fig MIP-1 peptides do not activate (A) SPRR-1 or (B) SPRR-3. Ca^2+^ responses of CHO cells expressing SPRR-1 or SPRR-3, challenged with 10 μM MIP-1 peptides, are shown relative (%) to the baseline (BSA cell medium without peptide). For SPRR-1 a single calcium response is plotted whereas for SPRR-3 the average of two independent experiments is presented together with the SD.
(TIF)

S4 Fig (A-B) Labeled confocal Z-stack projections showing expression of an *sprr-2p*::*sprr-2*::*SL2*::*gfp* transgene in adult hermaphrodites. Asterisk marks fluorescence in the intestine resulting from the co-injection marker *elt-2p*::*mCherry*. Scale bars are 15 μm. D, dorsal; V, ventral; A, anterior; P, posterior. (A) Overlap with OH4165 strain, marking ASE and AWC red, shows co-localization in ASE neurons. (B) DiI staining (red) validates *sprr-2* expression in ASI and AWB neurons.
(TIF)

S5 Fig (A) Blocking translation (cycloheximide) or transcription (actinomycin D) during conditioning does not impair wild-type animals to learn a positive association between 100 mM NaCl and the presence of food (p>0.05). Individual CIs are indicated as blue dots. Boxplots indicate 25th (lower boundary), 50th (line), and 75th (upper boundary) percentiles. Whiskers show minimum and maximum values. Statistical comparisons by one-way ANOVA and Tukey post-hoc test (n ≥ 8). (B) Salt chemotaxis behavior of wild-type and crh-1 mutants in response to increasing NaCl concentrations. Two-way ANOVA statistical analysis showed that salt chemotaxis of crh-1 mutants did not significantly differ from wild-type worms at 0.1, 10, 100 and 500 mM NaCl whereas there was a significant difference at 200 mM (***p<0.001). Mean chemotaxis indices with SD are plotted.
(TIF)

S1 Table Strains used in this study and corresponding figures. (DOCX)

S2 Table List of plasmids generated in this study and primers used for cloning. (DOCX)

S3 Table Overview of associative learning paradigms used in this study and corresponding figures. (DOCX)
