# Supplementary figures and images for: Myoinhibitory peptide signaling modulates aversive gustatory learning in Caenorhabditis elegans
Source: PLoS Genet. 2019 Feb 19;15(2):e1007945. doi: 10.1371/journal.pgen.1007945 (PMC6380545; doi:10.1371/journal.pgen.1007945)

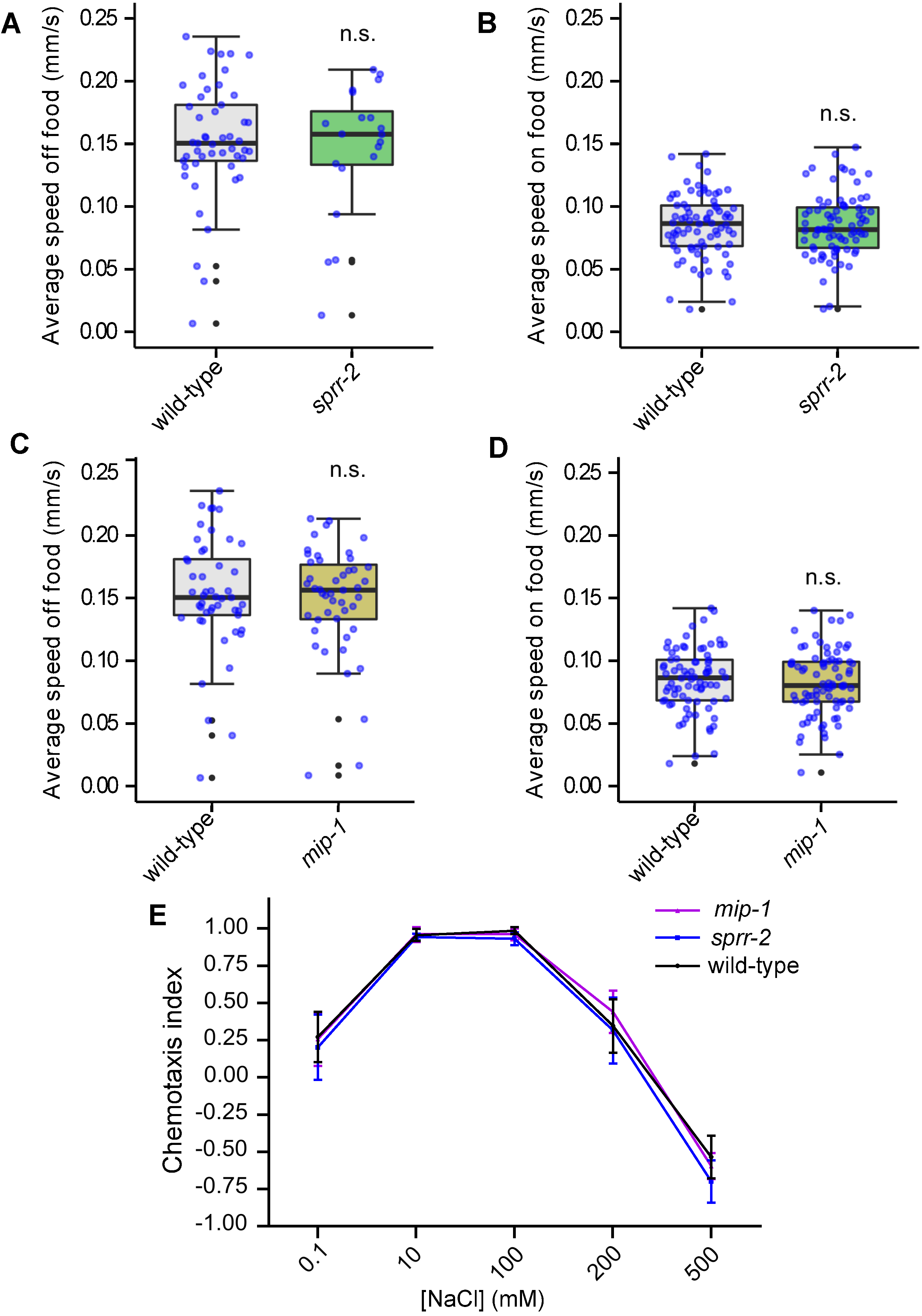

Supplement: S1 Fig — (A-D) Average speeds of individual (n ≥ 20) worms off food (A & C) or on an OP50 bacterial lawn (B & D) are scattered as blue dots. Boxplots indicate 25th (lower boundary), 50th (line), and 75th (upper boundary) percentiles. Whiskers show the minimum and maximum values. Outliers are indicated as black dots. Data were analyzed by one-way ANOVA with Tukey post-hoc test. The average speed of sprr-2 and mip-1 mutants is not significantly different (p>0.05) from that of wild-type animals. (E) Mock-conditioned mip-1 and sprr-2 mutants show normal salt chemotaxis behavior to increasing NaCl concentrations. Two-way ANOVA statistical analysis did not reveal any differences in NaCl chemotaxis behavior of mip-1 and sprr-2 mutants as compared to wild-type animals. Mean chemotaxis indices with SD are plotted for wild-type animals and mip-1 and sprr-2 mutants for NaCl concentrations ranging from 0.1 to 500 mM. (TIF) [file pgen.1007945.s002.tif]

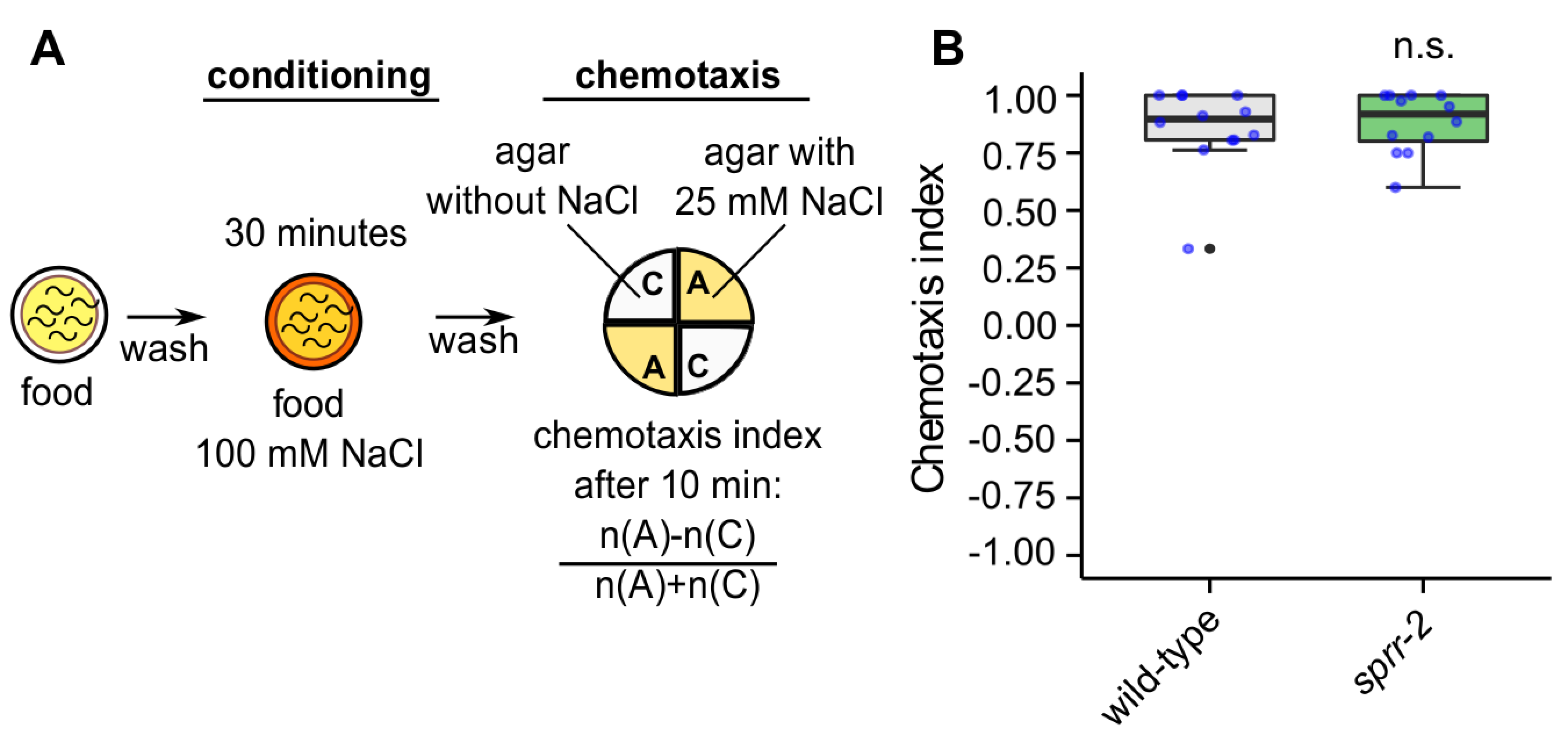

Supplement: S2 Fig — (A) NaCl chemotaxis behavior after conditioning salt with food. Synchronized 1-day adult C. elegans were conditioned on NaCl-containing plates in the presence of bacterial food. Chemotaxis behavior to NaCl was then tested on a quadrant plate. The CI is calculated from the number of worms that are present on quadrants with (A) or without (C) NaCl after 10 minutes. (B) NaCl chemotaxis behavior of sprr-2 mutants conditioned with salt in the presence of food is not significantly different (p>0.05) from the behavior of wild-type animals. Data were analyzed by one-way ANOVA with Tukey post-hoc test (n ≥ 10). Boxplots indicate 25th (lower boundary), 50th (line), and 75th (upper boundary) percentiles. Whiskers show the minimum and maximum values. Outliers are indicated as black dots. Individual CIs are plotted as blue dots. (TIF) [file pgen.1007945.s003.tif]

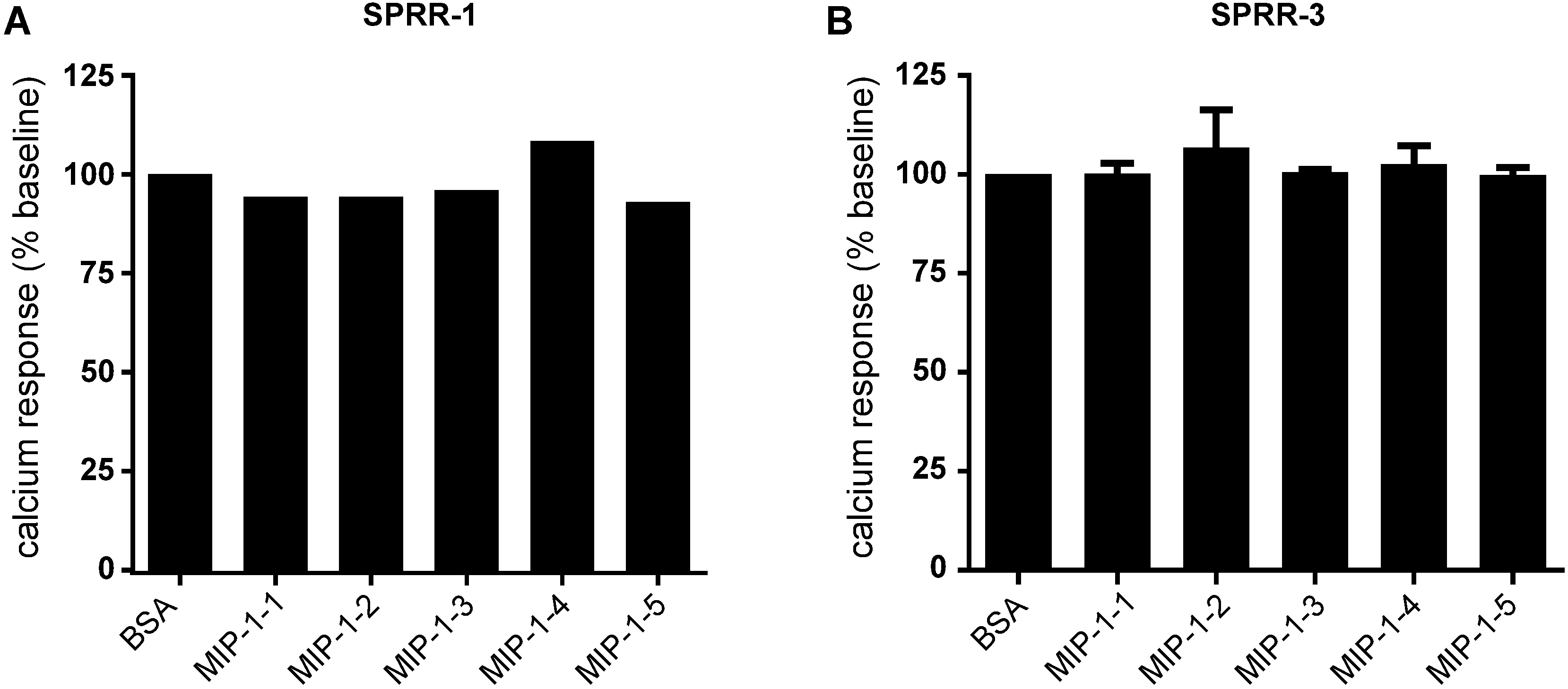

Supplement: S3 Fig — Ca2+ responses of CHO cells expressing SPRR-1 or SPRR-3, challenged with 10 μM MIP-1 peptides, are shown relative (%) to the baseline (BSA cell medium without peptide). For SPRR-1 a single calcium response is plotted whereas for SPRR-3 the average of two independent experiments is presented together with the SD. (TIF) [file pgen.1007945.s004.tif]

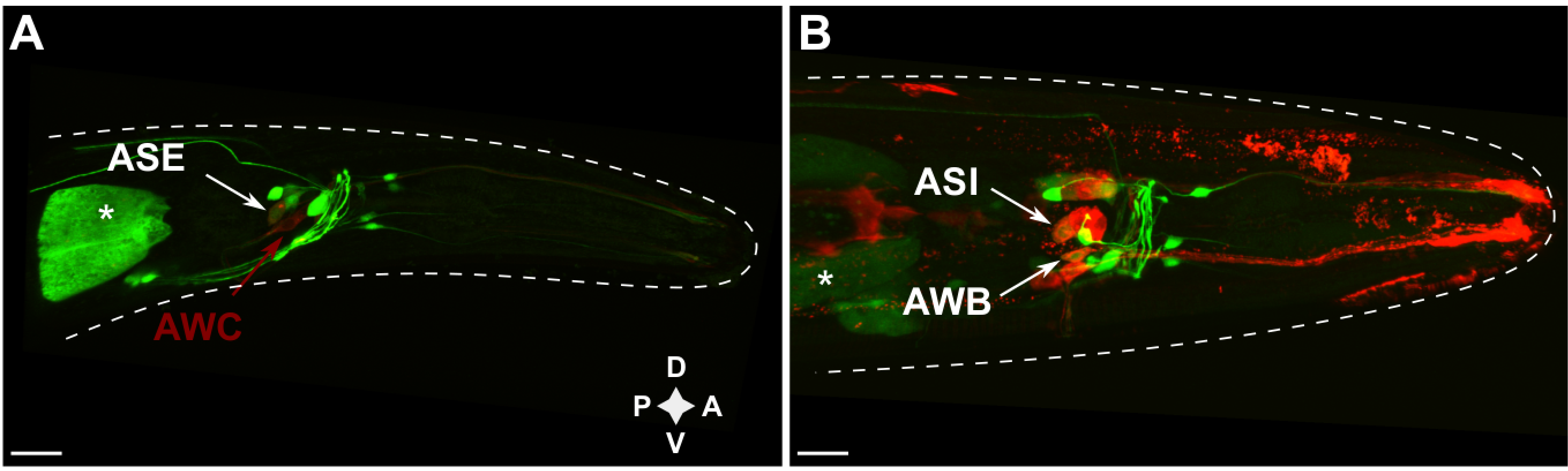

Supplement: S4 Fig — Asterisk marks fluorescence in the intestine resulting from the co-injection marker elt-2p::mCherry. Scale bars are 15 μm. D, dorsal; V, ventral; A, anterior; P, posterior. (A) Overlap with OH4165 strain, marking ASE and AWC red, shows co-localization in ASE neurons. (B) DiI staining (red) validates sprr-2 expression in ASI and AWB neurons. (TIF) [file pgen.1007945.s005.tif]

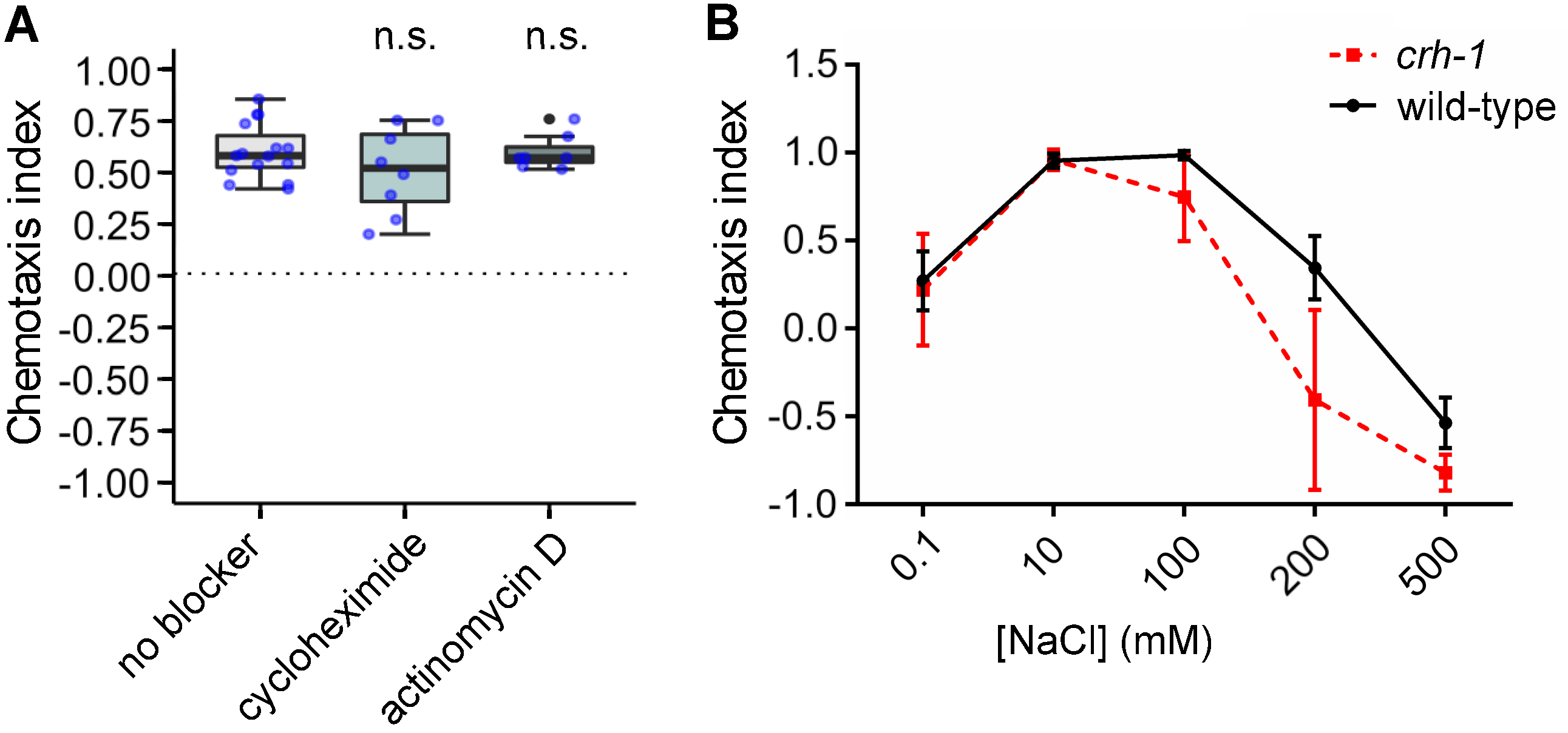

Supplement: S5 Fig — (A) Blocking translation (cycloheximide) or transcription (actinomycin D) during conditioning does not impair wild-type animals to learn a positive association between 100 mM NaCl and the presence of food (p>0.05). Individual CIs are indicated as blue dots. Boxplots indicate 25th (lower boundary), 50th (line), and 75th (upper boundary) percentiles. Whiskers show minimum and maximum values. Statistical comparisons by one-way ANOVA and Tukey post-hoc test (n ≥ 8). (B) Salt chemotaxis behavior of wild-type and crh-1 mutants in response to increasing NaCl concentrations. Two-way ANOVA statistical analysis showed that salt chemotaxis of crh-1 mutants did not significantly differ from wild-type worms at 0.1, 10, 100 and 500 mM NaCl whereas there was a significant difference at 200 mM (***p<0.001). Mean chemotaxis indices with SD are plotted. (TIF) [file pgen.1007945.s006.tif]
