# Supplementary material for: Myoinhibitory peptide signaling modulates aversive gustatory learning in Caenorhabditis elegans
Source: PLoS Genet. 2019 Feb 19;15(2):e1007945. doi: 10.1371/journal.pgen.1007945 (PMC6380545; doi:10.1371/journal.pgen.1007945)
Supplement: S1 Table — (DOCX) [file pgen.1007945.s007.docx]

S1 Table. Strains used in this study and corresponding figures

| Strain | Genotype | Figures |
| --- | --- | --- |
| N2 | wild-type Bristol variety | Fig 1B-C, Fig 3A-B, Fig 4E, Fig 5A-H, Fig 6A-C, S1A-E Fig, S2B Fig, S5A-B Fig |
| CF1038 | *daf-16(mu86)* | Fig 6A |
| FK223 | *egl-4(ks60)* | Fig 5G |
|  | *ins-1(nr2091)* | Fig 6C |
| MH534 | *mek-2(ku114); let-60(n1046)* | Fig 5G |
| VC220 | *cmk-1(ok287); gkDf56 Y102A5C.36(gk3558)* | Fig 5G |
| YT17 | *crh-1(tz2)* | Fig 5G-H, S5B Fig |
| LSC1002 | *sprr-1(tm3658)* | Fig 1B |
| LSC1010 | *sprr-3(tm3625)* | Fig 1B |
| LSC1013 | *sprr-2(tm3668)* | Fig 1B-C, Fig 3A, Fig 4E, Fig 5B-C, E, and H, Fig 6A-C, S1A-B & E, S2B Fig |
| LSC1100 | *nlp-38(ok2330)* | Fig 3A-B, Fig 5B and D-E, S1C-E Fig |
| LSC1203 | *lstEx931*[pSM-*sprr-2p::sprr-2 cDNA::sl2::gfp*), 25 ng/µL + pCIM02 (*unc-122p::dsRED*), 50 ng/μL] | Fig 4A |
| LSC1271 & LSC1273 | *sprr-2(tm3668); lstEx805*[pSM-*sprr-2p::sprr-2 cDNA::sl2::gfp*, 10 ng/µL + pCIM02 (*unc-122p::dsRED*), 50 ng/μL] | Fig 1C, Fig 5C |
| LSC1318 | *lstEx804*[pSM-*sprr-2p::sprr-2 gDNA::sl2::gfp*, 25 ng/µL+ pCIM01 (*elt-2p::mCherry*), 50 ng/μL] | Fig 4B-C, S4B Fig |
| LSC1326 | *nlp-38(ok2330); lstEx967*[pSM-*mip-1p::mip-1 gDNA::sl2::gfp*, 50 ng/µL + pCIM01 (*elt-2p::mCherry*), 50 ng/μL] | Fig 3B |
| LSC1313 & LSC1314 | *lstEx848*[pSM-*mip-1::mip-1 gDNA::sl2::gfp*, 25 ng/µL + pCIM01 (*elt-2p::mCherry)*, 50 ng/μL] | Fig 3C-E |
| LSC1512 | LSC1203; OH4165 *otIs151*[*ceh-36p::rfp + rol-6(su1006)*]; *otEx2416*[*gcy-21p::gfp; unc-122p::gfp*] | S4A Fig |
| LSC1527 | LSC1100; LSC1013 | Fig 3A, Fig 5E |
| LSC1638 | *sprr-2(tm3668)*; *lstEx883*[*gpa-4p::sprr-2 cDNA::sl2::gfp*, 10 ng/µL + pCIM02 *(unc-122p::dsRED)*, 50 ng/μL] | Fig 4E |
| LSC1676 & LSC1677 | LSC1013; CF1038 | Fig 6A |
| LSC1679  & LSC1680 | *sprr-2(tm3668)*; *lstEx904*[pSM-*gcy-5p::sprr-2 cDNA::sl2::gfp*, 10 ng/µL + pCIM02 *(unc-122p::dsRED)*, 50 ng/μL] | Fig 4E |
| LSC1682 & LSC1683 | *sprr-2(tm3668)*; *lstEx907*[*gcy-7p::sprr-2 cDNA::sl2::gfp*, 10 ng/µL + pCIM02 *(unc-122p::dsRED)*, 50 ng/μL] | Fig 4E |
| LSC1746 | LSC1013; RB759 | Fig 6B |
| LSC1751 | LSC1013; YT17 | Fig 5H |
| LSC1775 | *nlp-38(ok2330); lstEx997*[pSM-*mip-1::mip-1 gDNA::sl2::gfp*, 10 ng/µL + pCIM03 (*unc-122p::GFP)*, 50 ng/μL] | Fig 5D |
| LSC1778 | LSC1013; *ins-1(nr2091)* | Fig 6C |
| RB759 | *akt-1(ok525)* | Fig 6B |
|  |  |  |
