# Supplementary material for: Myoinhibitory peptide signaling modulates aversive gustatory learning in Caenorhabditis elegans
Source: PLoS Genet. 2019 Feb 19;15(2):e1007945. doi: 10.1371/journal.pgen.1007945 (PMC6380545; doi:10.1371/journal.pgen.1007945)
Supplement: S2 Table — (DOCX) [file pgen.1007945.s008.docx]

**S2 Table.** List of plasmids and PCR products generated in this study and primers used

| Plasmid | Fragment | Forward primer (5' - 3') | Reverese primer (5' - 3') |
| --- | --- | --- | --- |
| Plasmid: pSM-*sprr-2p::sprr-2 cDNA::sl2::gfp* | *sprr-2*  *sprr-2p* | ttggctagcgtcgacggtacATGAACTACGAAGTTTATTG  gactgggcgcgcctctagagCAATAAGTTTCGAGTGTTTTGAGG | gtaggatgagacagcggtacTTAATCACTCCTATCCATC  gtcctttggccaatcccgggGCCCTCCACAATTTCACC |
| Plasmid: pSM-*sprr-2p::sprr-2 gDNA::sl2::gfp* | *sprr-2*  *sprr-2p* | ttggctagcgtcgacggtacATGAACTACGAAGTTTATTG  gactgggcgcgcctctagagCAATAAGTTTCGAGTGTTTTGAGG | gtaggatgagacagcggtacTTAATCACTCCTATCCATC  gtcctttggccaatcccgggGCCCTCCACAATTTCACC |
| Plasmid: pSM-*mip-1p::mip-1 gDNA::sl2::gfp* | *nlp-38/mip-1*  *nlp-38p/mip-1p* | ttggctagcgtcgacggtacATGCAGCTGATACACTTTATTG  gactgggcgcgcctctagagAACTTAGGAAAATACTTCGTAAGCATGCC | gtaggatgagacagcggtacTTATCTTCCCCAAAGACC  gtcctttggccaatcccgggATTTCTTTTCTTTTTGCGCC |
| Plasmid: pSM-*gcy-5p::sprr-2 cDNA::sl2::gfp* | *sprr-2*  *gcy-5p* | ttggctagcgtcgacggtacATGAACTACGAAGTTTATTG  gactgggcgcgcctctagagTCTTACATTTTGACACGAATTC | gtaggatgagacagcggtacTTAATCACTCCTATCCATC  gtcctttggccaatcccgggTTTTCATCAGAATAAGTAATTTTTC |
| Plasmid: pCDNA3.1/V5-His-TOPO-*sprr-1 cDNA* | *sprr-1* | CACCAATTCAAGTTGGAGCCCGAAAG | TGAGACCAATTTGATGTGAGA |
| Plasmid: pCDNA3.1/V5-His-TOPO-*sprr-2 cDNA* | *sprr-2* | CACCATGAACTACGAAGTTTATTGC | TTAATCACTCCTATCCATCGTTG |
| Plasmid: pCDNA3.1/V5-His-TOPO-*sprr-3 cDNA* | *sprr-3* | CACCATGACCGATTTGGAGATTTG | CTACGAATTTTCTCTTCGAATCTTC |
| PCR product: *gpa-4p::sprr-2 cDNA::sl2::gfp* | *sprr-2*  *gpa-4p*  *Fusion gpa-4p and sprr-2* | ATGAACTACGAAGTTTATTGC  TGACAGAAGACAGAGACTCGA  TGACAGAAGACAGAGACTCGA | GAGCTGCATGTGTCAGAGGT  GCCGCAATAAACTTCGTAGTTCATAATGAATAAGTGGCTCTACC  AGGAAACAGTTATGTTTGGTA |
| PCR product: *gcy-7p::sprr-2 cDNA::sl2::gfp* | *sprr-2*  *gcy-7p*  *Fusion gcy-7p and sprr-2* | ATGAACTACGAAGTTTATTGC  GAAAAGGTTTTAGATTTCGA  GAAAAGGTTTTAGATTTCGA | GAGCTGCATGTGTCAGAGGT  GCCGCAATAAACTTCGTAGTTCATTCTTATGCTAAACTGGCAGACA  AGGAAACAGTTATGTTTGGTA |
