# Supplementary material for: Myoinhibitory peptide signaling modulates aversive gustatory learning in Caenorhabditis elegans
Source: PLoS Genet. 2019 Feb 19;15(2):e1007945. doi: 10.1371/journal.pgen.1007945 (PMC6380545; doi:10.1371/journal.pgen.1007945)
Supplement: S3 Table — (DOCX) [file pgen.1007945.s009.docx]

S3 Table. Overview of associative learning paradigms used in this study and corresponding figures.

| Learning paradigm | Described in | Conditioning | Chemotaxis assay | | Figures |
| --- | --- | --- | --- | --- | --- |
| Gustatory plasticity | [11-13] | 15 minutes in buffer with 100 mM NaCl in the absence of bacterial food | Quadrant | Fig 1A-C  Fig 3A-B, Fig 4E | |
| Gustatory plasticity mocked trained | [11] | 15 minutes in buffer without NaCl in the absence of bacterial food | Quadrant | Fig 1A-B, S1E Fig  S5B Fig | |
| Taste associative learning negative association: Salt avoidance learning | [8-10] | 6 hours conditioning on NGM plates with 100 mM NaCl in the absence of bacterial food | Grandient | Fig 5A-H  Fig 6A-C | |
| Taste associative learning positive association | [8] | 6 hours conditioning on NGM plates with 100 mM NaCl in the presence of bacterial food | Gradient | Fig 5A-B  S5A Fig | |
| Gustatory plasticity on food | [12] | 30 minutes conditioning on NGM plates with 100 mM NaCl in the presence of bacterial food | Quadrant | S2A-B Fig | |
